# The Nrf2 inhibitor brusatol is a potent antitumour agent in an orthotopic mouse model of colorectal cancer

**DOI:** 10.18632/oncotarget.25497

**Published:** 2018-06-05

**Authors:** Jonathan P. Evans, Boleslaw K. Winiarski, Paul A. Sutton, Robert P. Jones, Lorenzo Ressel, Carrie A. Duckworth, D. Mark Pritchard, Zhi-Xiu Lin, Fretwell L. Vicky, Elizabeth M. Tweedle, Eithne Costello, Christopher E. Goldring, Ian M. Copple, B. Kevin Park, Daniel H. Palmer, Neil R. Kitteringham

**Affiliations:** ^1^ Department of Molecular and Clinical Cancer Medicine, Institute of Translational Medicine, University of Liverpool, Liverpool, United Kingdom; ^2^ MRC Centre for Drug Safety Science, Department of Molecular and Clinical Pharmacology, Institute of Translational Medicine, University of Liverpool, Liverpool, United Kingdom; ^3^ Department of Veterinary Pathology, Institute of Veterinary Science, University of Liverpool, Liverpool, United Kingdom; ^4^ Department of Cellular and Molecular Physiology, Institute of Translational Medicine, University of Liverpool, Liverpool, United Kingdom; ^5^ School of Chinese Medicine, Faculty of Medicine, The Chinese University of Hong Kong, Hong Kong, People’s Republic of China; ^6^ Clatterbridge Cancer Centre, Liverpool, United Kingdom

**Keywords:** colorectal cancer, Nrf2, brusatol, irinotecan, orthotopic syngeneic model

## Abstract

Nrf2 is a transcription factor that regulates cellular stress response and irinotecan-metabolising pathways. Its aberrant activity has been reported in a number of cancers, although relatively few studies have explored a role for Nrf2 in colorectal cancer (CRC). This study assessed the expression of Nrf2 in patient CRC tissues and explored the effect of Nrf2 modulation alone, or in combination with irinotecan, in human (HCT116) and murine (CT26) cell lines *in vitro* and in an orthotopic syngeneic mouse model utilising bioluminescent imaging. Using a tissue microarray, Nrf2 was found to be overexpressed (p<0.01) in primary CRC and metastatic tissue relative to normal colon, with a positive correlation between Nrf2 expression in matched primary and metastatic samples. *In vitro* experiments in CRC cell lines revealed that Nrf2 siRNA and brusatol, which is known to inhibit Nrf2, decreased viability and sensitised cells to irinotecan toxicity. Furthermore, brusatol effectively abrogated CRC tumour growth in subcutaneously and orthotopically-allografted mice, resulting in an average 8-fold reduction in luminescence at the study end-point (p=0.02). Our results highlight Nrf2 as a promising drug target in the treatment of CRC.

## INTRODUCTION

Colorectal cancer (CRC) continues to be the second leading cause of cancer-related death in the Western World, with the worst outcomes in patients with metastatic disease (mCRC) [1]. Neoadjuvant chemotherapy can bring patients with mCRC to resection, while in the adjuvant setting it can treat systemic disease. First-line treatment varies with geographical location, in the US the FOLFIRI regimen (5-flurouracil, leucovorin and Irinotecan) predominates. In the UK, irinotecan-based regimens are more commonly employed in the management of patients with advanced disease who have failed with oxaliplatin based therapy. Chemotherapy-response varies significantly, disease progression is common, while debilitating side-effects or systemic toxicity can limit treatment [2]. The development of novel therapies is essential and is achieved through the identification of drug-targetable molecular pathways involved in cancer cell survival or chemo-resistance.

Nuclear factor-erythroid 2-related factor 2 (Nrf2) is a transcription factor that, amongst other roles, regulates the expression of drug metabolising and antioxidant genes and confers cytoprotection against cellular stress. Under basal conditions Nrf2 is sequestered by kelch-like ECH-associated protein 1 (Keap1), which promotes its degradation in the proteasome. Studies have implicated Nrf2 in cancer cell survival and chemo-resistance, with an increasing number of reports of its constitutive over-expression in a number of malignancies [3, 4]. As a result, there is interest in the therapeutic potential of modulating Nrf2 activity in the context of CRC and other cancers.

Nrf2 plays an important role in drug disposition, with potential consequences for the efficacy of chemotherapeutic agents, particularly irinotecan. Irinotecan is a water-soluble pro-drug converted to the active metabolite SN-38, which inhibits topoisomerase I [5]. Nrf2 regulates the expression of the human carboxylesterases [6], required for the hydrolysis of irinotecan to SN-38 [7–9], in addition to regulating the expression of UGT1A1, responsible for deactivation of SN-38 by glucuronidation [10, 11]. Any therapeutic strategy involving Nrf2 manipulation needs to consider both the direct impact on tumour development and any indirect, and potentially undesirable, effects due to altered metabolism or transport of co-administered chemotherapeutics.

The availability of pharmacological inducers and inhibitors of Nrf2 offers the possibility of modulating this pathway in the clinical setting. The synthetic triterpenoid, methyl-2-cyano-3,12-dioxooleano-1,9-dien-28-oate (CDDO-me), is a potent inducer of Nrf2. CDDO-me is a derivative of oleanolic acid demonstrated to increase Nrf2 activity [12]. Brusatol is a naturally occurring quassinoid extracted from the aerial parts of the *Brucea javanica* plant that has been shown to inhibit Nrf2 [13–15]. Fruit and seed oil from the *Brucea javanica* plant have traditionally been used in Chinese medicine [16], whilst brusatol has been shown to overcome chemoresistance in both *in vitro* and *in vivo* models of lung cancer [15, 17].

The effect of Nrf2 modulation in the management of CRC is unpredictable, particularly in the context of irinotecan cytotoxicity, due to complex interactions between drug metabolism and cell survival pathways. With approximately 50% of irinotecan converted to SN-38 in the liver, understanding the interaction between tumour and host is key, making *in vivo* assessment essential [9]. Here, we provide evidence for the elevated expression of Nrf2 in CRC tumour tissue, compared with matched normal colonic mucosa, and demonstrate a positive correlation between Nrf2 expression in matched CRC primary and metastatic tissue. We demonstrate that modulation of Nrf2 can sensitise CRC cells to the cytotoxic effects of irinotecan *in vitro*, and that brusatol can inhibit tumour growth in a syngeneic orthotopic mouse model of CRC. Our findings highlight Nrf2 as a potential drug target in the treatment of CRC.

## RESULTS

### Nrf2 is upregulated in CRC patient samples

**Figure 1:**
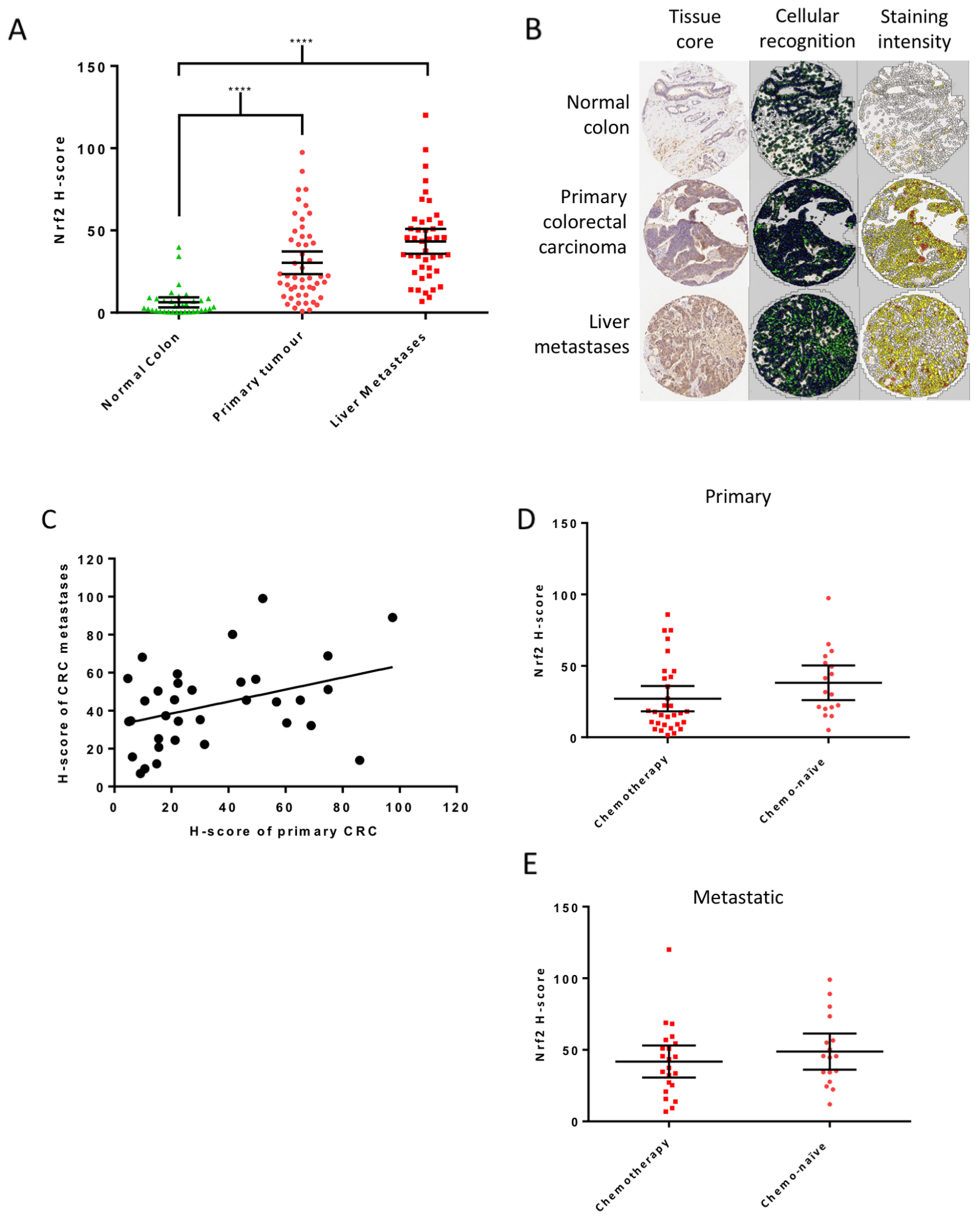
Nrf2 expression in patient samples **(A)** Mean Nrf2 expression, measured by the calculation of H-scores, was significantly higher in primary (H-score = 30) and metastatic (H-score = 43) tumour tissue than normal colon (H-score 6, Kruskal-Wallis with Dunn’s multiple comparisons test). **(B)** Examples of tissue cores as analysed by Tissue Studio v.2.0 showing cellular recognition (green for cytoplasm and blue for nucleus) and Nrf2 staining intensity as assigned by the software; white represents negative, yellow weakly positive, orange moderately positive and red strongly positive cells. **(C)** A positive correlation in Nrf2 expression was confirmed between matched primary tumour and liver metastases in patient samples (r=0.4, p=0.03, Pearson’s R correlation). Comparison of H-scores in chemo-naïve and treated patients showed no significant difference in Nrf2 expression in primary **(D)** or metastatic tissue **(E)** (Mann-Whitney test). Graphs display mean with 95% confidence interval.

Fifty-nine patients with mCRC were included in the tissue microarray, 50 (85%) with cores of primary CRC, 43 (73%) with cores of liver metastases and 34 (58%) with normal colon available. Matched primary and metastatic samples were available for 34 (58%) patients. Clinicopathological details of the patients are included in Table 1. Nrf2 expression was significantly higher in primary and metastatic CRC samples than in normal colon (Figure 1A and 1B). Nrf2 expression did not alter significantly with sex, T stage or N stage (Supplementary Table 1). A positive correlation was noted between the Nrf2 H-scores of matched primary and metastatic samples (Figure 1C), indicating that Nrf2 expression in the metastasis reflects that of the primary tumour. There was no difference in Nrf2 expression in the primary (Figure 1D) or metastatic samples (Figure 1E) of chemo-naïve patients when compared to those who received neoadjuvant chemotherapy, suggesting the observed high expression of Nrf2 was not simply a marker of increased cellular stress induced by chemotherapy.

**Table 1:** Clinicopathological variables for patients contributing samples to the tissue microarray stained for Nrf2 expression

Variable		Primary Tumour	Liver Metastases	Normal Colon	P value
Median age (range)		78 (40-98)	78 (40-98)	79 (40-98)	p = 0.834 (ANOVA)
Gender (%)	Male	35 (70)	29 (67)	23 (68)	p = 0.958 (Chi-Square)
	Female	15 (30)	14 (33)	11 (32)	
T stage (%)	1	0	0	0	p = 0.911 (Chi-Square)
	2	8 (16)	4 (9)	4 (13)	
	3	33 (66)	31 (72)	24 (71)	
	4	9 (18)	8 (19)	6 (18)	
N stage (%)	0	14 (28)	11 (26)	11 (23)	p = 0.486 (Chi-Square)
	1	26 (52)	25 (58)	21 (62)	
	2	10 (20)	7 (16)	2 (6)	
Chemotherapy (%)	Yes	31 (62)	22 (51)	22 (65)	p = 0.597 (Chi-Square)
	No	17 (34)	17 (40)	11 (32)	
	Unknown	2 (4)	4 (9)	1 (3)	
**Total**		50	43	34	

There were no statistically significant differences between patients variables included in the analysis of available tissue cores.

### Inhibition of Nrf2 reduces CRC cell viability and proliferative capacity

**Figure 2:**
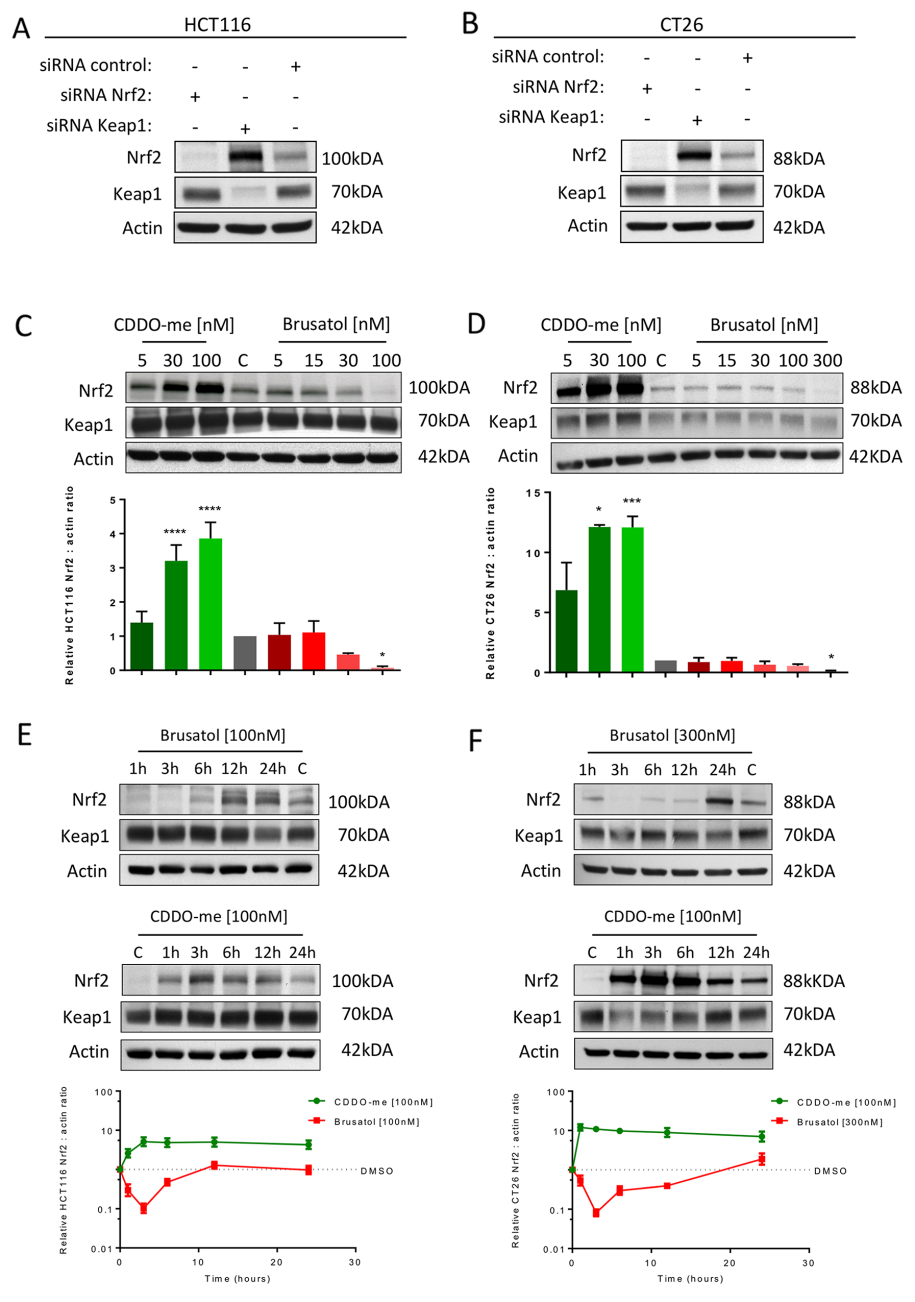
Western blot confirmation of Nrf2 modulation in HCT116 **(A)** and CT26 **(B)** cells using siRNA targeting *Nrf2* or *Keap1* and pharmacologically with brusatol or CDDO-me in HCT116 **(C)** and CT26 **(D)** cells. Significant Nrf2 inhibition required 100nM and 300nM of brusatol in the HCT116 and CT26 cell lines respectively while significant Nrf2 induction was demonstrated at 30nM of CDDO-me in both cell lines (one-way ANOVA with Holm-Sidak’s multiple comparison test). The effect of brusatol on Nrf2 was transient in HCT116 **(E)** and CT26 **(F)** cells with the maximum effect at three hours. CDDO-me induction resulted in upregulation of Nrf2 for greater than 24 hours. All graphs display mean data with error bars representing standard deviation. C= 0.5% DMSO vehicle control.

To explore the effect of Nrf2 modulation on CRC cell growth, studies were conducted in human (HCT116) and murine (CT26) CRC-derived cell lines. Levels of Nrf2 were modulated either by genetic silencing of Nrf2 or Keap1 utilising siRNA (Figure 2A and 2B) or pharmacologically with the potent inducer CDDO-me or with brusatol, which is known to inhibit Nrf2 (Figure 2C and 2D). A higher concentration of brusatol was required to significantly inhibit Nrf2 in CT26 cells compared with HCT-116 cells. In both cell lines, and consistent with previous reports [13, 15], brusatol-mediated inhibition of Nrf2 was transient, with maximum inhibition observed at three hours (Figure 2E and 2F). The effects of Nrf2 inhibition on the downstream target Nqo1 are displayed in Supplementary Figure 2.

**Figure 3:**
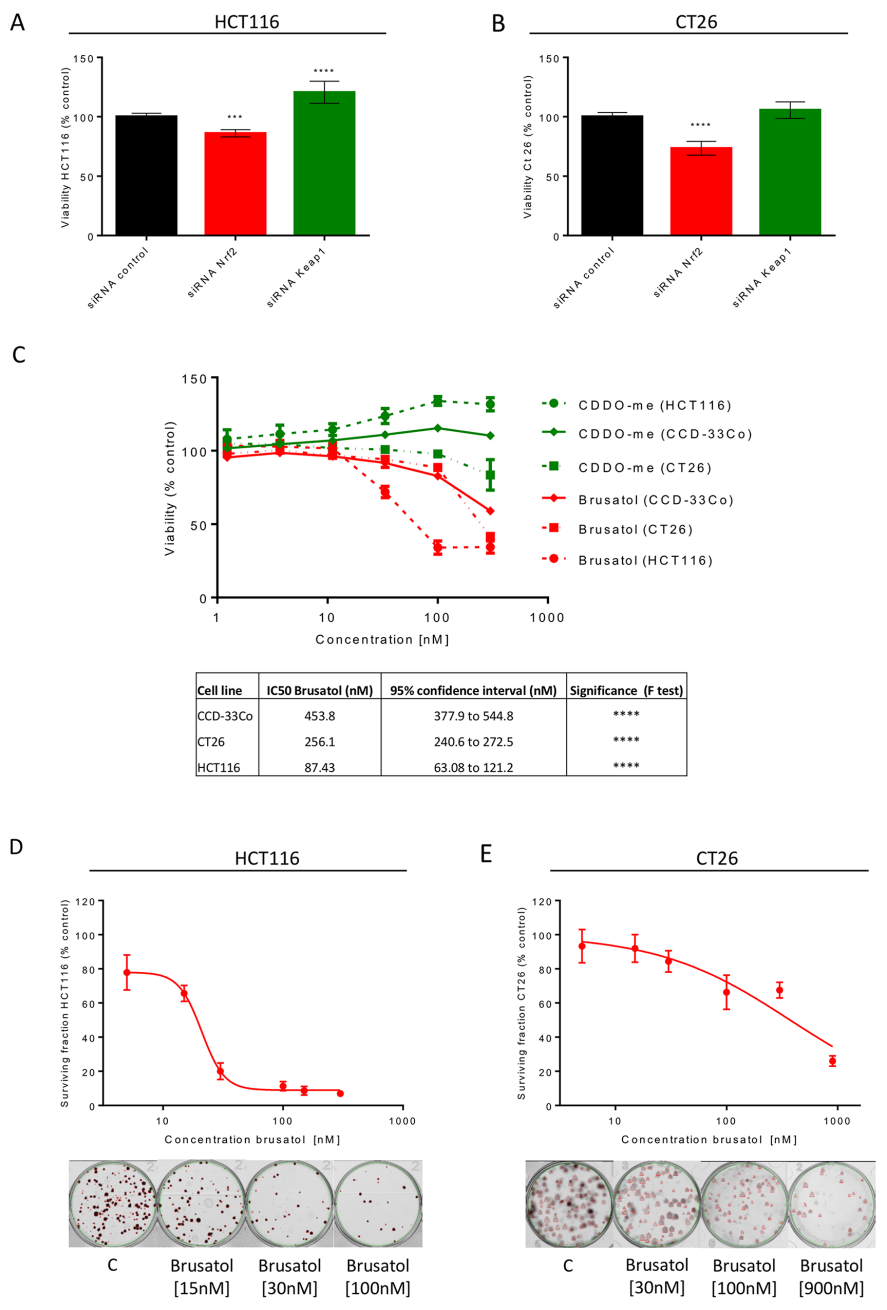
Cell viability assessment in cell lines following Nrf2 modulation siRNA inhibition of Nrf2 in HCT116 **(A)** and CT26 cells **(B)** caused a significant decrease in viability (one-way ANOVA with Holm-Sidak’s multiple comparison test). **(C)** Brusatol reduced cell viability in a concentration-dependent manner in all cell lines, but to a lesser extent in benign CCD-33Cocolonic cells, as reflected in the relative IC50 values (extra sum-of-squares *F* test). CDDO-me induction of Nrf2 caused a small increase in proliferation of HCT116 and CCD-33Co cells. Assessment of reproductive integrity following exposure to brusatol confirmed inhibition of colony formation, represented by calculation of surviving fractions expressed as a percentage of untreated cells, in HCT116 **(D)** and CT26 cells **(E)**. All graphs display mean data with error bars representing standard deviation. C= 0.5% DMSO vehicle control.

siRNA inhibition of Nrf2 resulted in a 14% and 23% reduction in cell viability in HCT116 (Figure 3A) and CT26 (Figure 3B) cells respectively, establishing a role for Nrf2 in the survival of CRC cells. The opposite trend was noted for activation of Nrf2 through Keap1 inhibition in HCT116 cells, with an increase in viability of 21% (Figure 3A); there was a non-significant 7% increase in the viability of CT26 cells transfected with Keap1 siRNA (Figure 3B). The modulation of the known Nrf2 target Nqo1 confirmed the altered activity of the Nrf2 pathway in cells transfected with siRNA (Supplementary Figure 2). Brusatol also reduced viability in both cancer cell lines, while in HCT116 cells CDDO-me slightly increased cell viability in a concentration-dependent manner (Figure 3C). The effect of CDDO-me was not noted in the CT26 cells (Figure 3C), which have higher basal expression of Nrf2. In benign human CCD-33Co colonic cells, the effects of CDDO-me and brusatol on viability were less marked than in CRC cells (Figure 3C). Finally, to assess the effect of brusatol on CRC cell proliferation, clonogenic assays were performed. Brusatol reduced colony formation and the surviving fraction in both CRC cell lines, with IC50 values of 21nM (95% CI, 19-23nM) and 373nM (95% CI, 277-502nM) in HCT116 and CT26 cells respectively, suggesting a reduced proliferative capacity following brusatol treatment (Figure 3D and 3E).

### Nrf2 inhibition sensitises CRC cells to irinotecan

**Figure 4:**
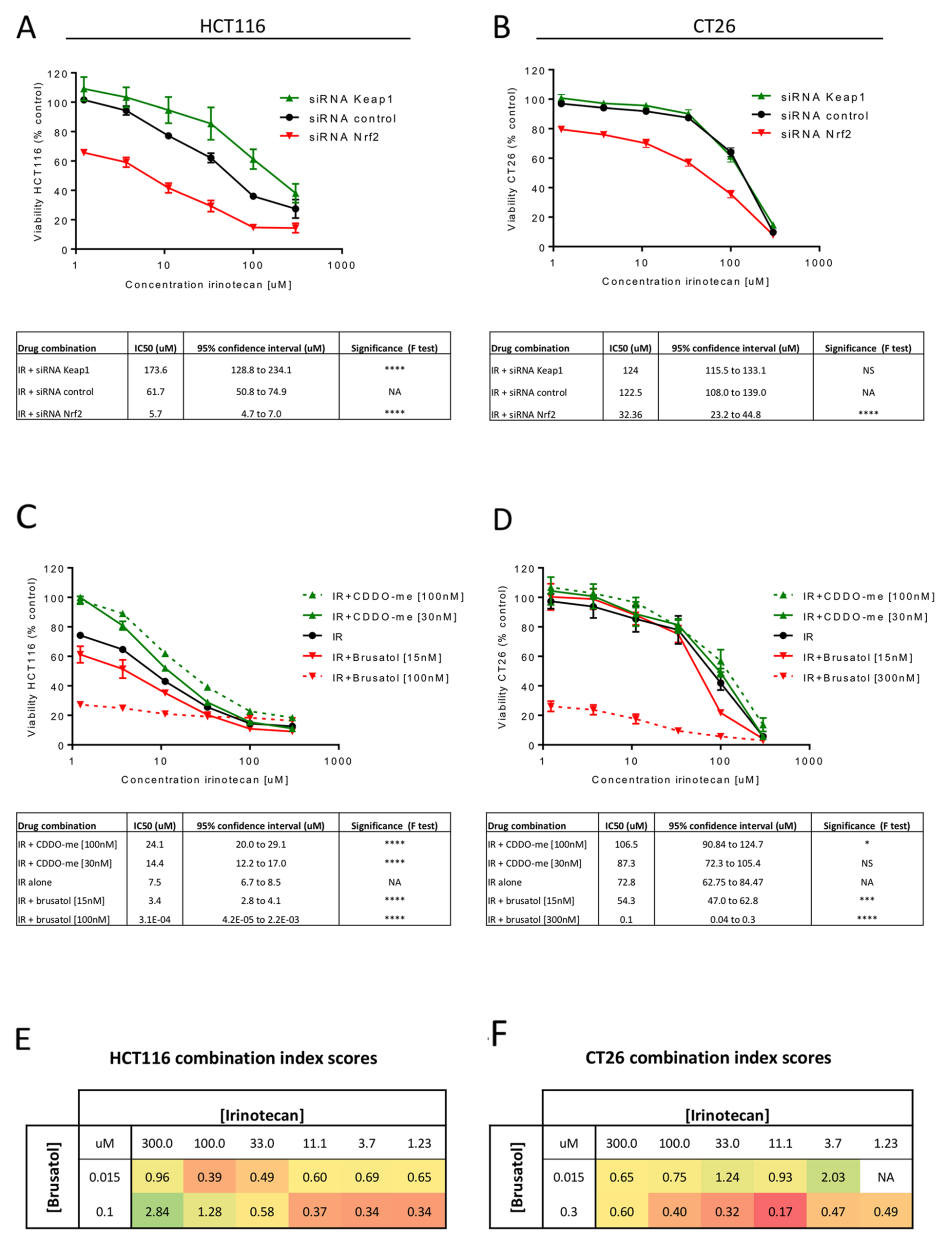
Assessment of cell viability for the combination of irinotecan with Nrf2 modulation Nrf2 inhibition using siRNA significantly increased the cytotoxicity of irinotecan in HCT116 **(A)** and CT26 **(B)** cells as reflected by the decrease IC50 values when compared to treatment with irinotecan alone (extra sum-of-squares *F* test). The cytoprotective effect of overexpression of Nrf2 by KEAP1 inhibition was less marked in the CT26 (B) cell line than in HCT116 (A). Pharmacological modulation of Nrf2 with brusatol and CDDO-me in combination with irinotecan showed the same trends as seen with siRNA transfection in both HCT116 **(C)** and CT26 **(D)** cells. Calculated combination indexes for treatment with irinotecan and brusatol confirm drug synergy in HCT116 **(E)** and CT26 **(F)** cells across a range of concentrations with red signifying a higher degree of synergy. All graphs display mean data with error bars representing standard deviation. IR = irinotecan.

Having demonstrated that modulation of Nrf2 affects CRC cell viability and proliferative capacity, it was important to ensure that Nrf2 modulation did not negatively impact the efficacy of chemotherapeutics. The effect of siRNA or pharmacological modulation of Nrf2 on the cytotoxicity of irinotecan was assessed in HCT116 and CT26 cells. Both Nrf2 siRNA (Figure 4A and 4B) and brusatol (Figure 4C and 4D) significantly decreased the IC50 of irinotecan in the CRC cell lines, signifying increased sensitivity to irinotecan following Nrf2 depletion. The cytoprotective effect of Nrf2 activation was again more marked in the HCT116 cell line than in CT26 cells (Figure 4C and 4D), potentially indicating a biological limit to the protective effect of activation of the Nrf2 pathway. Combination indices demonstrated a synergistic enhanced cytotoxic response when brusatol and irinotecan were co-incubated with the CRC cell lines (Figure 4E and 4F). In contrast, when brusatol and irinotecan were applied to benign CCD-33Co cells the effect was found to be mostly additive, with little drug synergy evident (Supplementary Figure 3). Co-treatment of the CRC cell lines with brusatol also reduced the IC50 of 5-flurouracil, although drug synergy was achieved at fewer concentrations than noted with irinotecan (Supplementary Figure 4), possibly due to a differential influence of Nrf2 on the metabolism of 5-flurouracil.

### Brusatol reduces CRC disease burden and improves the efficacy of irinotecan therapy *in vivo*

**Figure 5:**
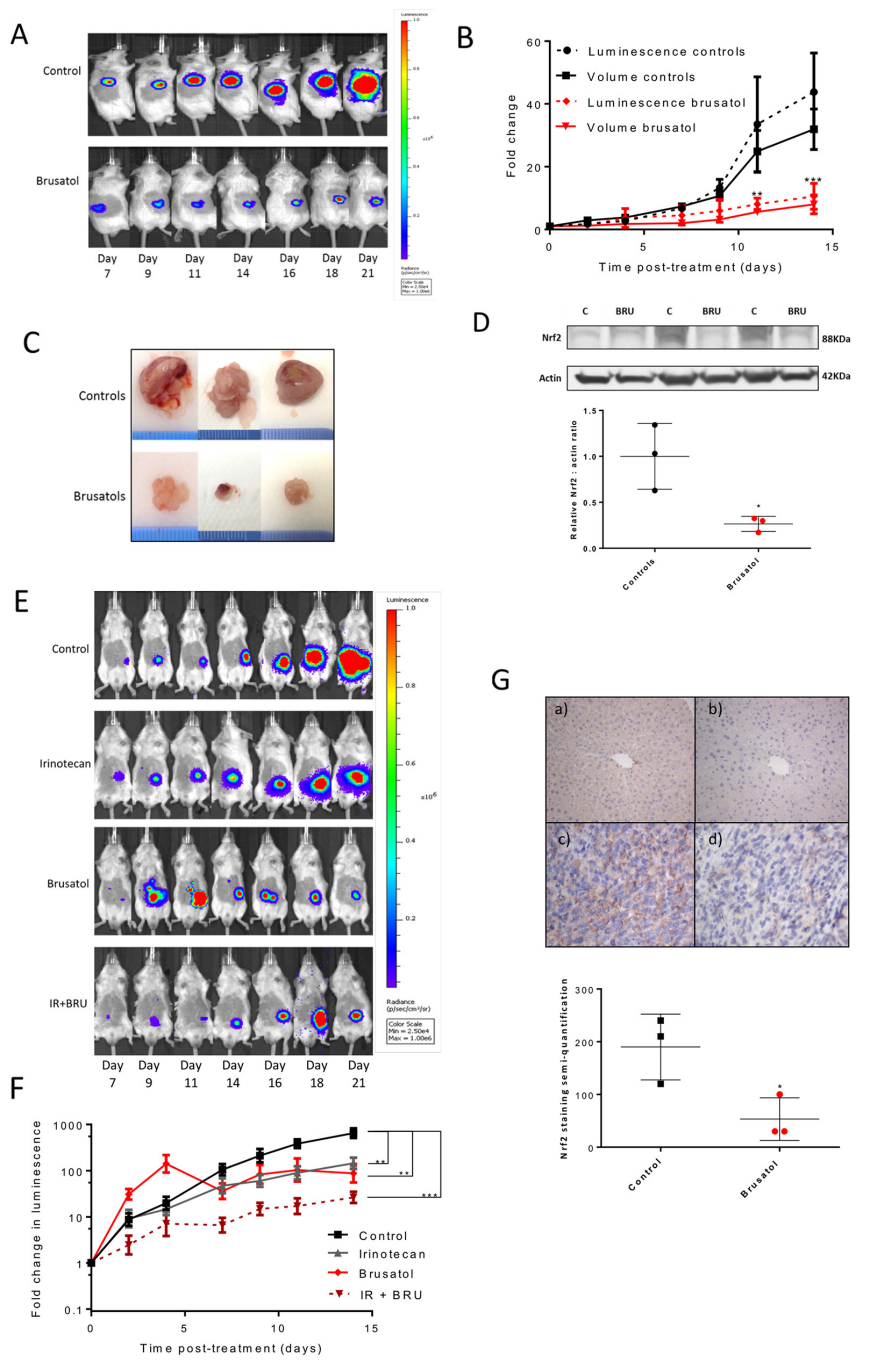
The effects of Nrf2 modulation on tumour growth *in vivo* **(A)** Example IVIS images of BALB/c mice from the brusatol treated and control groups after subcutaneous flank injection of the CT26lucA6c cell line. **(B)** Graphical display of the significant inhibition of tumour growth in mice treated with brusatol in comparison to vehicle treated controls (multiple *t*-tests, N=3/group, mean +/-SEM). **(C)** Photos of the flank tumours excised from mice (scale in 1mm increments) 21 days after implantation. **(D)** Western immunoblotting and densitometry confirmed significant inhibition of Nrf2 in the flank tumours excised from mice treated with brusatol (p=0.03, unpaired *t*-test, bar chart displays mean and standard deviation). **(E)** Example serial IVIS^®^ images of BALB/c mice orthotopically injected with the CT26lucA6c cell line with time from implantation of cells. **(F)** Data displayed graphically as fold change in luminescence from the first day of treatment. All treatments inhibited tumour growth significantly compared to mice in the control group (one-way ANOVA, N=8, graphs display mean +/- SEM). **(G)** Representative images from tissue stained for Nrf2 by IHC. Moderate to strong diffuse staining is demonstrated in the a) positive control of liver from BALB/c mice treated with CDDO-me. b) Livers from Nrf2 knockout mice were used as a negative control and were absent of Nrf2 staining. There was strong staining demonstrated in caecal tumours excised from mice in the c) control group and staining was reduced in the tumours from mice treated with d) brusatol. Graphical display of Nrf2 staining semi-quantification reveals significantly (p=0.04 unpaired *t*-test with Welch’s correction) reduced Nrf2 staining semi-quantification in brusatol treated mice (N=3, graph displays mean +/- SD). C = control, BRU = brusatol, IR = irinotecan.

Based on the *in vitro* findings, the therapeutic potential of brusatol was assessed *in vivo* following the injection of luciferase-expressing CT26 cells, either into the flank or orthotopically into the caecal wall, of BALB/c mice. Prior to *in vivo* studies, brusatol dose-response curves were generated *in vitro* using the luciferase-expressing CT26 cells to confirm phenotypic similarity to the parent population (Supplementary Figure 5). Treatment with brusatol markedly reduced the growth of flank tumours, as represented by fold change in luminescence and tumour volume *in vivo* (p<0.01; Figure 5A and 5B), and tumour volume at necropsy (Figure 5C). Nrf2 expression in excised tumour tissue was reduced by 74% in mice treated with brusatol compared to controls (p=0.03; Figure 5D). A significant reduction in tumour growth rate with brusatol treatment (represented by fold change in luminescence) was also noted in the syngeneic orthotopic CRC model (p<0.01; Figure 5E and 5F). Notably, in this model there was a trend towards enhanced anti-tumour efficacy in mice treated with a combination of brusatol and irinotecan, compared with each treatment in isolation (Figure 5E and 5F; Supplementary Figure 6). Indeed, at the final time-point the fold change in tumour size was significantly different in mice treated with a combination of irinotecan and brusatol when compared with irinotecan alone (mean fold change = 26.4 versus 144.1, p=0.04, unpaired *t*-test with Welch correction). Whilst no adverse effects were noted in mice receiving brusatol, several mice in the control group began to exhibit tumour-related symptoms including ascites, weight loss and gastrointestinal obstruction, limiting the study end-point. IHC staining of randomly selected caecal tumours demonstrated reduced Nrf2 expression in mice treated with brusatol (mean Nrf2 staining score = 53.3) when compared with the saline-treated controls (mean Nrf2 staining score = 190; Figure 5G).

## DISCUSSION

Following the discovery of Nrf2 as a master regulator of cellular defence [18, 19], its early therapeutic prospects were perceived to principally lie in chemoprevention, due to its ability to counteract the potentially damaging effects of environmental chemicals, oxidative stress and ionising radiation [20]. Mounting evidence revealed a darker side to Nrf2 in cancer, with many tumour types displaying elevated levels of the transcription factor, often due to loss-of-function mutations in the Keap1 gene that promote Nrf2-driven transcriptional activity [21]. Along with a small number of other studies in CRC patients [22, 23], the tissue microarray performed here confirms up-regulation of Nrf2 in primary and metastatic CRC tissue. Our data also agree with previous studies indicating that higher Nrf2 expression contributes to chemo-resistance in CRC cell lines, possibly due to the up-regulation of cytoprotective mechanisms such as glutathione and other antioxidant pathways, or through increased expression of drug export proteins and metabolising enzymes [24–26]. Increased Nrf2 activity in CRC may occur for a number of reasons: hypermethylation of the *Keap1* promoter region is common in CRC cell lines, suppressing its mRNA expression [27]; somatic mutations in Keap1 were found in 8% of CRCs, resulting in loss of inhibitory function [21]; and activation of the KRAS-MEK-ERK pathway has been associated with high levels of Nrf2 expression in lung and pancreatic cancer [17, 28]. Oncogenic mutations in *KRAS* occur in 32% of CRC tumours [29] and both CRC cell lines used in this study are *KRAS* mutants [30, 31]. These findings imply increased Nrf2 expression conveys a survival benefit to cancer cells, which may become dependent on increased Nrf2 activity.

This is the first study to demonstrate that brusatol can suppress tumour growth and enhance the chemotherapeutic effect of irinotecan in mouse models of CRC. Brusatol has been shown to inhibit Nrf2 and enhance chemosensitivity in a number of cancer cell lines [13, 15, 32]. However, recent work has indicated that brusatol blocks cap-dependent and -independent translation and provokes decreases in the levels of several short-lived proteins, in addition to Nrf2 [14, 33]. These findings are consistent with earlier work showing that quassinoids bind to the 80S ribosome, inhibiting protein elongation [34]. Therefore, whilst the inhibition of Nrf2 may partly underlie the ability of brusatol to sensitise CRC cell lines to irinotecan and supress tumour growth *in vivo*, it is possible that effects on other critical mediators may also play a role. Alternatively, Chio *et al.* recently showed that Nrf2 regulates the activity of the translational machinery, with genetic disruption of Nrf2 in pancreatic cancer organoids causing defects in epidermal growth factor receptor signalling upstream of cap-dependent translation, as well as oxidation of specific translational regulatory proteins [35]. Therefore, the inhibition of Nrf2 by brusatol may exacerbate its effect on translation of other proteins, although the transient nature of its effect on Nrf2 noted here and previously [13, 15] imply that this is not a perpetual cycle. As new inhibitors with greater specificity to Nrf2 emerge [36] it will be important to confirm the value of modulating Nrf2 *per se* in the context of CRC and other cancers.

Despite its reported impact on general protein synthesis, brusatol was well tolerated in our animal studies, with no adverse effects noted. Consistent with this, Lu *et al.* recently showed that daily administration of brusatol for 28 days could inhibit the growth of pancreatic cancer xenografts in mice in the absence of any changes in body weight or tissue injury biomarkers [32]. Extracts of the *Brucea javanica* plant, from which brusatol is derived, have been used in Chinese medicine to treat diseases including malaria and gastric ulcers [16]. Whilst this further supports the tolerability of natural quassinoids, to the best of our knowledge the exposure to brusatol in these settings has not been established through pharmacokinetic studies. Therefore, more work is necessary to fully establish the safety and therapeutic potential of brusatol in patients, yet this study provides proof-of-concept that brusatol has beneficial effects in CRC. One approach to limiting possible systemic off-target effects of brusatol is to deliver it to tumours through the use of drug carriers [37] or chemo-embolisation [38]. In the context of mCRC, where irinotecan-based regimens may be the last available management option, the enhancement of irinotecan-based chemotherapy could overcome chemo-resistance, improve treatment responses and allow dose reduction, which in turn could minimise adverse reactions and improve the wellbeing of patients.

## MATERIALS AND METHODS

### Immunohistochemical analysis in patient samples

NHS Research Ethics Committee and Research and Development approval was obtained for work on patient samples. Tissue microarrays were constructed from formalin-fixed paraffin embedded (FFPE) primary tumour, liver metastases and normal adjacent colonic mucosa from patients with metastatic CRC. Tissue cores were stained for Nrf2 expression using the primary antibody for Nrf2 SC-722 (Santa Cruz, CA; 1:50 dilution; two hours) and semi-quantification of protein expression performed by Tissue Studio v.2.0 (Definiens AG, Munich, Germany). H-scores were calculated as described by Shousha [39]. See supplementary methods for additional details.

### Cell culture

Human CRC (HCT116, European Collection of Cell Cultures) cells, murine BALB/c derived CRC (CT26, American Type Culture Collection) cells and normal immortalised human colonic cells (CCD-33Co, American Type Culture Collection) were cultured in DMEM, RPMI1640 and EMEM media respectively. Media were supplemented with 10% fetal bovine serum, 100 units/ml penicillin G and 100 μg/ml streptomycin. The luminescent CT26lucA6c clone was co-cultured with the selection antibiotic G-418 (Promega, Madison, WI).

### Luminescent cells

CT26 cells were stably transfected with the pGL4.51 (Promega) plasmid using Lipofectamine^®^ 2000 (ThermoFisher, Waltham, MA) as per the manufacturer’s recommendations and selected via supplementation of the media with G-418 (Promega, Madison, WI). The most luminescent clone was passaged in the flank of an immune-competent BALB/c mouse, producing CT26lucA6c cells.

### Genetic modulation of Nrf2

siRNA targeting human *NRF2* (siGenome D-003755-05, NM_001145413, Dharmacon, Lafayette, CO) or *KEAP1* (siGenome D-012453-03, Nm_012289, Dharmacon); or murine *Nrf2* (siGenome MQ-040766-00, NM_010902, Dharmacon) or *Keap1* (FlexiTube Mm_Keap1_7, NM_001110305, Qiagen, Venio, Netherlands) were used to modulate Nrf2 in the CRC cell lines. Transient transfections were performed using Lipofectamine^®^ RNAiMAX (ThermoFisher), as per the manufacturer’s instructions. A non-targeting scrambled siRNA was used as a control in all experiments. Cells were transfected with siRNA for 48 h prior to application of chemotherapeutics or lysis for immunoblotting.

### *In vitro* drug dosing and cell viability assay

Stock concentrations of all drugs, including irinotecan (J&H Chemical, Shanghai, China), 5-FU (Sigma-Aldrich, St. Louis, MI), CDDO-me (Cayman chemicals, Ann Arbor, MI) and brusatol (obtained as described previously [13]) were prepared in dimethyl sulfoxide (DMSO), serially-diluted and added to media. To assess the effect of Nrf2 modulation on chemo-sensitivity, cells were either transfected with siRNA 48 h prior to the application of chemotherapeutics or co-dosed with CDDO-me or brusatol. Cell viability was assessed by introducing CellTiter 96^®^ AQueous One Solution Cell Proliferation Assay (Promega, UK) to wells 2 h prior to analysis of light absorbance (490 nm) with a Varioskan™ reader. Results were calculated as a percentage of vehicle (0.5 % DMSO) treated cells. For western immunoblotting, cells were treated with CDDO-me or brusatol and lysed at the indicated time points for time-course experiments, or after three hours for dose-response analysis. All experiments were performed in triplicate on at least three occasions.

### Western immunoblotting

Western immunoblotting was undertaken on 40 micrograms of cell lysate and tumour tissue homogenate were loaded in Laemmli buffer (BioRad, Hercules, CA) on Mini-PRTOEAN^®^ TGX™ Precast Gels (BioRad, Hercules, CA). After electrophoresis, proteins were transferred to nitrocellulose membranes and subject to immunoblot analysis using primary antibodies for Nrf2 (Proteintech, Manchester, UK; 1:1000 dilution, 16396-1-AP), NQO1 (Abcam, Cambridge, UK; 1:2000 dilution, ab28947) and actin (Abcam, Cambridge, UK; 1:10 000 dilution, ab6726). Semi-quantitative densitometry was performed by Image-J v1.47 with samples normalised to actin.

### Colony formation assay

Cells were exposed to vehicle control or brusatol for 48 hours and then re-plated at 200 live cells/well on collagen coated plates. After 7-10 days colonies were fixed with methanol, stained with 0.5% crystal violet and counted using GelCount™ (Oxford Optronix, Oxford, UK). The surviving fraction (SF) was calculated as a percentage of the vehicle control using the method described by Franken et al [40].

### *In vivo* investigation

All animal experiments were performed under a UK Home Office approved project licence in a licenced establishment. BALB/c (Charles River, UK) mice were housed in a specific-pathogen-free environment on a 12-hour light-dark cycle with free access to food and water. Brusatol treatment (2 mg/kg, see supplementary figures 1 for regimens) was compared to vehicle control (1% DMSO in phosphate buffered saline)following the subcutaneous injection of 5×10^5^ CT26lucA6c cells. Mice were randomised to treatment arms seven days after the injection of cells, with calliper measurement and bioluminescence imaging used to quantify tumour progression. Bioluminescence imaging was performed using an IVIS Spectrum system (Perkin Elmer, Waltham, MA). Tumours were excised at the end of the study, homogenised in 0.1% sodium dodecyl sulfate / 0.5M triethylammonium bicarbonate buffer and subject to western immunoblotting.

An orthotopic syngeneic murine model of CRC was established by injecting 4×10^5^ CT26lucA6c cells into the caecal sub-serosa as described previously [41] (see Supplementary Figure 1 for technique). Bioluminescent imaging of mice was utilised for the assessment of disease burden and mice with detectable signal randomised to treatment regimens: vehicle control, brusatol alone (2mg/kg), irinotecan alone (20mg/kg) or irinotecan plus brusatol (for dosing regimens see Supplementary Figure 1). Luminescent signals were quantified in photons/second and tumour growth expressed as fold change in luminescence from the first imaging day (i.e. pre-treatment), with each mouse acting as its own control. At the end of the study mice were sacrificed and tumour tissue stored in 4% paraformaldehyde prior to paraffin embedding for histological and immunohistochemical analyses.

### IHC analysis of murine tumours

Sections of FFPE tumour blocks were stained for Nrf2 expression as described in the supplementary material. Livers from CDDO-me treated mice served as a positive control and from Nrf2 knockout mice a negative control [42]. Semi-quantification of Nrf2 expression was undertaken blinded to experimental conditions by multiplying the percentage of the Nrf2 positive cells by the Nrf2 stain intensity (1=mild; 2=moderate; 3=marked).

### Statistical analysis

GraphPad Prism^®^ 6 statistical software was utilised for dose-response analysis, calculation of inhibitory concentration (IC50) values and for comparisons of statistical significance. IC50 values were calculated by fitting a four-parameter log-concentration versus response curve to the data. Drug combinations were assessed for synergy using the Chou-Talalay method (combination index < 1 indicates synergy) and Compusyn software [43]. For mouse studies the slope of a line of best fit for each individual mouse was calculated as a surrogate marker of tumour growth rate. Growth rates for treatment groups were then compared to those in untreated controls with significance assessed by one-way ANOVA. SPSS statistics 21^®^ was used to analyse patient data. A Shapiro-Wilk normality test was used to select an appropriate statistical test. Statistical significance was assumed at a two-sided p value of <0.05 (^*^p<0.05, ^**^p<0.01, ^***^p<0.001, ^****^p<0.0001).

## SUPPLEMENTARY MATERIALS FIGURES AND TABLE


