# Correction: The Nrf2 inhibitor brusatol is a potent antitumour agent in an orthotopic mouse model of colorectal cancer

**DOI:** 10.18632/oncotarget.26625

**Published:** 2019-01-18

**Authors:** Jonathan P. Evans, Boleslaw K. Winiarski, Paul A. Sutton, Robert P. Jones, Lorenzo Ressel, Carrie A. Duckworth, D. Mark Pritchard, Zhi-Xiu Lin, Vicky L. Fretwell, Elizabeth M. Tweedle, Eithne Costello, Christopher E. Goldring, Ian M. Copple, B. Kevin Park, Daniel H. Palmer, Neil R. Kitteringham

**Affiliations:** ^1^ Department of Molecular and Clinical Cancer Medicine, Institute of Translational Medicine, University of Liverpool, Liverpool, United Kingdom; ^2^ MRC Centre for Drug Safety Science, Department of Molecular and Clinical Pharmacology, Institute of Translational Medicine, University of Liverpool, Liverpool, United Kingdom; ^3^ Department of Veterinary Pathology, Institute of Veterinary Science, University of Liverpool, Liverpool, United Kingdom; ^4^ Department of Cellular and Molecular Physiology, Institute of Translational Medicine, University of Liverpool, Liverpool, United Kingdom; ^5^ School of Chinese Medicine, Faculty of Medicine, The Chinese University of Hong Kong, Hong Kong, People’s Republic of China; ^6^ Clatterbridge Cancer Centre, Liverpool, United Kingdom

**This article has been corrected:** The correct Author name is given below:

**Vicky L. Fretwell**

Original article: Oncotarget. 2018; 9:27104-27116. https://doi.org/10.18632/oncotarget.25497

