# Supplementary material for: The Nrf2 inhibitor brusatol is a potent antitumour agent in an orthotopic mouse model of colorectal cancer
Source: Oncotarget. 2018 Jun 5;9(43):27104–16. doi: 10.18632/oncotarget.25497 (PMC6007465; doi:10.18632/oncotarget.25497)
Supplement: Supplementary file 1 [file oncotarget-09-27104-s001.pdf]

The Nrf2 inhibitor brusatol is a potent antitumour agent in an orthotopic mouse model of colorectal cancer

SUPPLEMENTARY MATERIALS AND METHODS

Immunohistochemical analysis in patient samples

Tissue microarrays were constructed using a tissue microarrayer (Beecher Instruments Incorporated, Sun Prairie, WI) with 0.6mm cores retrieved and transferred into the recipient block in triplicate. After de-waxing in xylene, rehydration with ethanol solutions and blocking with 3% hydrogen peroxide, antigens were retrieved and slides incubated with the primary antibody for Nrf2 (Santa Cruz, CA; SC-722; 1:50 dilution; two hours) and horseradish peroxidase conjugated secondary antibody (Dako UK Ltd, Cheshire, UK; E0433; 1:200; 30 minutes). Following incubation with the Vectastain Elite® ABC reporter system (Vectorlabs, Burlingame, CA), slides were developed with diaminobenzidine tetrahydrochloride (DAB) and counterstained with haematoxylin. Stained sections of the TMA were scanned using the AperioScanscope (Leica Biosystems, Newcastle, UK) and semi-quantification of protein expression performed with Tissue Studio v.2.0 (Definiens AG, München, Germany). H-scores for cores were calculated as described by Shousha [1] by assigning staining intensities of negative, weak, moderate or high to cells with scoring as follows: H-score = (1 × percentage of cells scored 1) + (2 × percentage of cells scored 2) + (3 × percentage of cells scored 3).

Histology, immunohistochemical staining and analysis of murine tumours

From paraffin embedded tumour blocks, consecutive 4-microns thick sections were obtained and stained with haematoxylin eosin (HE) for morphological confirmation of the neoplastic process. Representative sections of the lesions were selected for immunohistochemistry. All tissue sections were placed in an automated staining system (Link 48 Immunostainer; Dako UK Ltd, Cheshire, UK) within which samples were deparaffinised, rehydrated and processed for epitope retrieval using computer-controlled antigen retrieval system (PT Link-Low pH; Dako UK Ltd, Cheshire, UK) according to the manufacturer's recommendations. Primary anti Nrf2 antibody (Proteintech, Manchester, UK; 1:100 dilution, antibody 16396-1-AP for 30 minutes) was applied and the anti-mouse/rabbit EnVision FLEX (Dako UK Ltd, Cheshire, UK) detection system used. Upon completion of the immunostaining, sections were counterstained with Mayer's haematoxylin. BALB/c mice liver treated with CDDO-me were used as a positive control, while livers from Nrf2 knockout mice were employed as a negative control [2]. Consecutive tumour sections incubated with non-immune rabbit serum served as technical negative control. Morphological evaluation and confirmation of tumour formation was performed by a board certified veterinary pathologist (LR) upon brightfield microscope examination of HE sections. Evaluation of Nrf2 expression was semi-quantitative (Nrf2 staining semi-quantification): for each tumour, the percentage of the Nrf2 positive tumour area was multiplied by the Nrf2 stain intensity (1=mild; 2=moderate; 3=marked). The semi-quantitative scoring was performed blinded to the experimental conditions.

REFERENCES

1. Shousha S. Oestrogen receptor status of breast carcinoma: Allred/H score conversion table. Histopathology. 2008; 53:346–347.
2. Itoh K, Chiba T, Takahashi S, Ishii T, Igarashi K, Katoh Y, Oyake T, Hayashi N, Satoh K, Hatayama I, Yamamoto M and Nabeshima Y. An Nrf2/small Maf heterodimer mediates the induction of phase II detoxifying enzyme genes through antioxidant response elements. Biochem Biophys Res Commun. 1997; 236:313–322.

Supplementary Table 1: Nrf2 expression did not vary significantly with gender, T stage or N stage in any of the tissue types

		Primary-tumour		Liver metastases		Normal colon	
**Variable**		**Mean H-score (95% CI)**	**P value**	**Mean H-score (95% CI)**	**P value**	**Mean H-score (95% CI)**	**P value**
Gender	Male	27 (20-36)	p = 0.265 (ANOVA)	39 (34-72)	p = 0.07 (ANOVA)	5 (2-9)	p = 0.403 (ANOVA)
	Female	36 (21-51)		53 (28-46)		8 (3-14)	
T stage	1	NA	p = 0.442 (ANOVA)	NA	p = 0.673 (ANOVA)	NA	p = 0.269 (ANOVA)
	2	39 (14-65)		54 (25-82)		2 (0-11)	
	3	27 (19-36)		43 (33-52)		6 (2-9)	
	4	33 (19-46)		41 (22-59)		11 (4-18)	
N stage	0	32 (16-49)	p = 0.556 (ANOVA)	50 (34-66)	p = 0.334 (ANOVA)	6 (0.2-12)	p = 0.986 (ANOVA)
	1	27 (18-36)		39 (29-49)		6 (2-10)	
	2	36 (19-54)		28 (24-75)		7 (0-20)	
**Totals**		30 (24-36)		43 (37-50)		6 (0-13)	

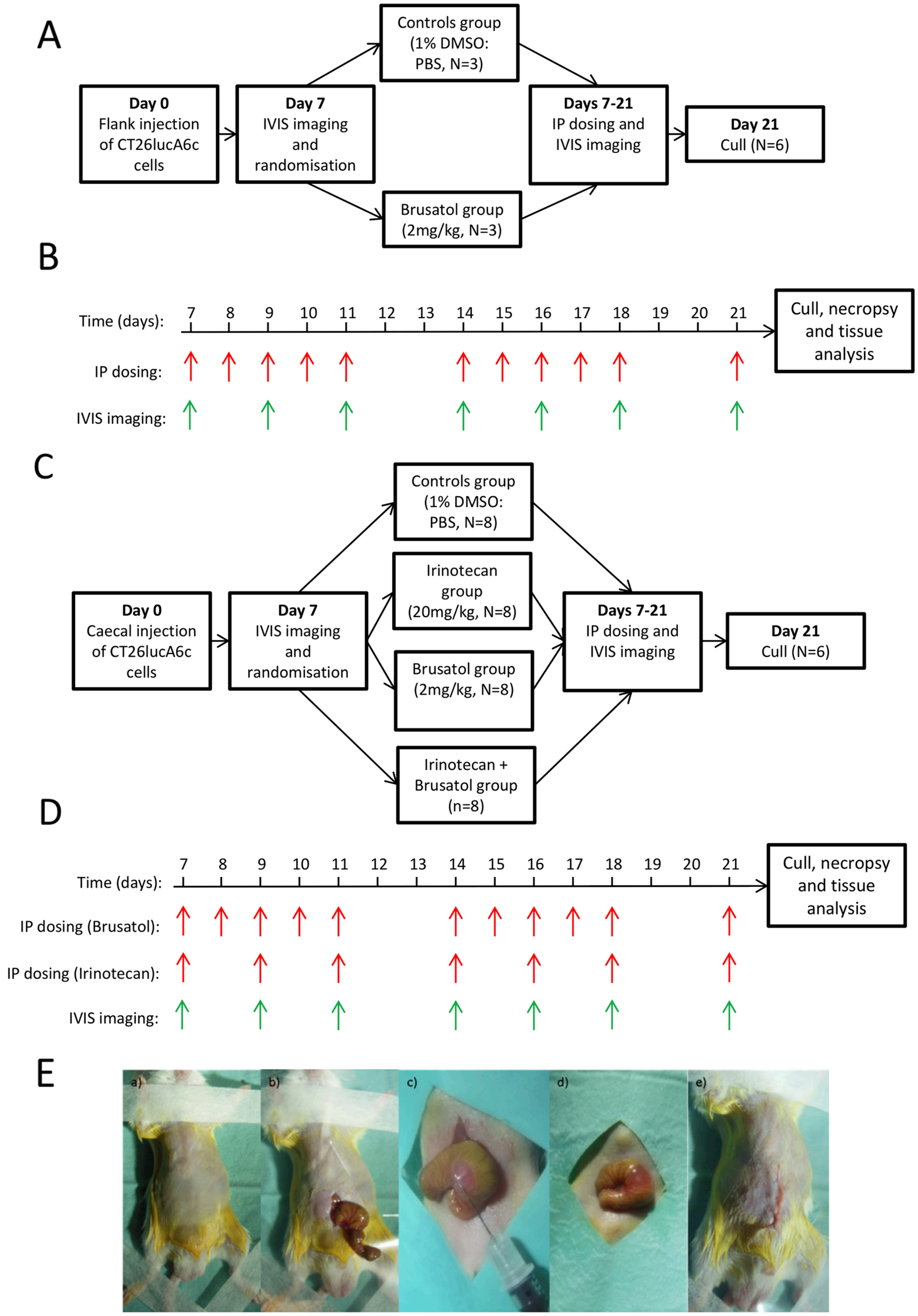

Supplementary Figure 1: **(A)** Murine study protocol used to test brusatol therapy after the flank injection of CT26lucA6c cells. **(B)** Displays the dosing schedule for control and brusatol treated groups, either control (0.1% DMSO in phosphate buffered saline) or brusatol (2mg/kg) were administered via intraperitoneal (IP) injection on the highlighted days (red arrow) with imaging conducted three times per week (green arrow) **(C)** Murine study protocol used to test brusatol therapy after the orthotopic injection of CT26lucA6c cells **(D)** Displays the dosing schedules for treatment regimens in the syngeneic orthotopic model of CRC using the CT26lucA6c cells. Control, brusatol, irinotecan (20mg/kg) or a combination of brusatol and irinotecan were administered via intraperitoneal (IP) injection on the highlighted days (red arrow) with imaging conducted three times per week (green arrow). **(E)** Images of the operative technique for the caecal injection of cells. a) Sterile draping was followed by b) midline laparotomy and caecal delivery. c) Injection of $5{\times}10^5$ CT26lucA6c cells in 1:4 PBS:Matrigel (Corning, UK) through a 28G needle raised d) a bleb of cell suspension in the caecal wall, following which the caecum was returned to the abdomen and e) the abdominal wall closed in two layers with a subcuticular 6-0 vicryl stitch.

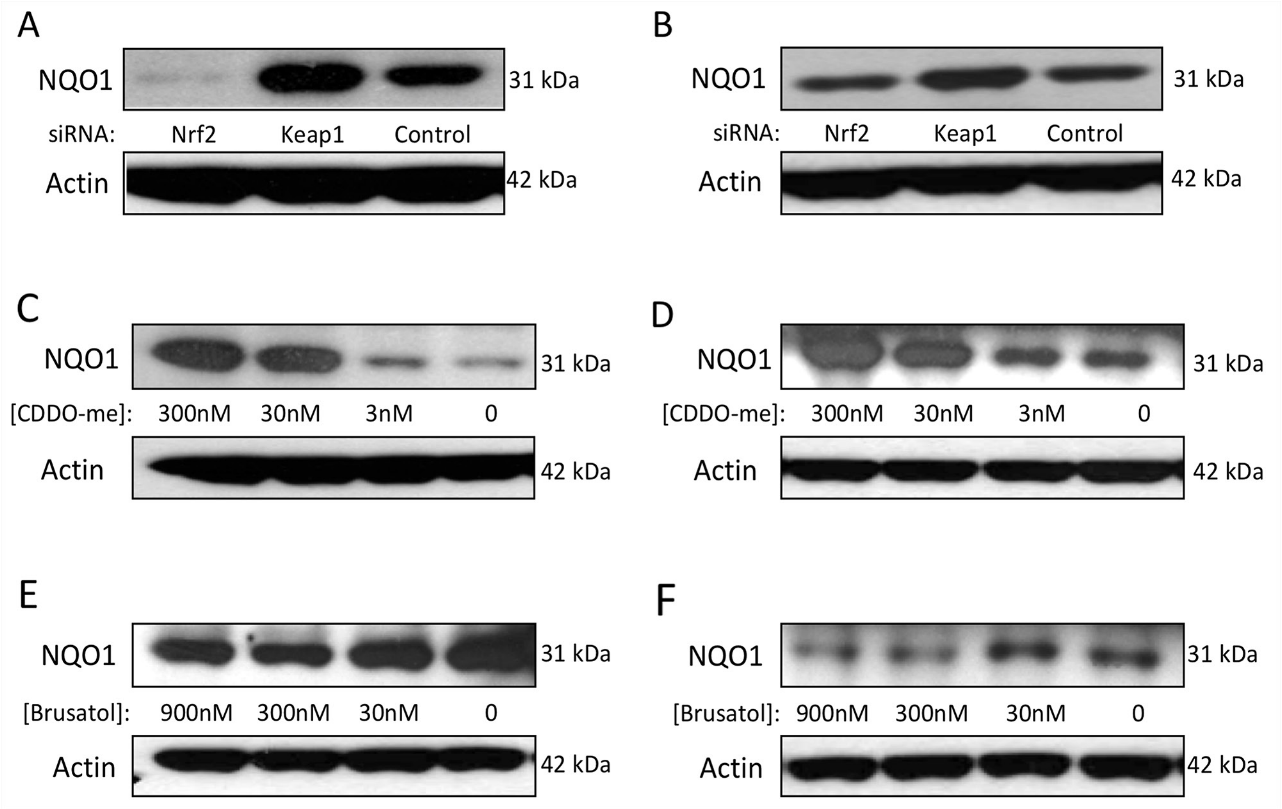

Supplementary Figure 2: Western blot confirmation of Nqo1 modulation in HCT116 **(A, C, E)** and CT26 **(B, D, F)** cells using siRNA targeting *Nrf2* or *Keap1*(A-B) and pharmacologically with CDDO-me (C-D) or brusatol (E-F).

A

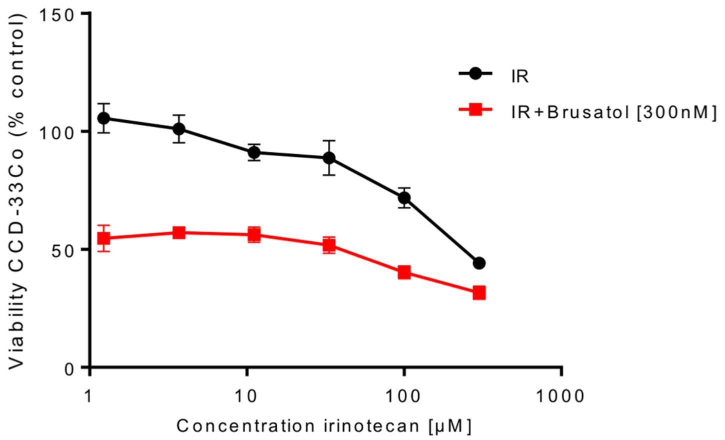

Drug combination	IC50 (μM)	95% confidence interval (μM)	Significance (F test)
IR alone	241.6	205 to 284.7	NA
IR + brusatol [300nM]	14.18	9.1 to 22.1	****

B

CCD-33Co combination index scores

	[Irinotecan]						
[Brusatol]	uM	300.0	100.0	33.0	11.1	3.7	1.23
	0.3	0.74	0.65	0.84	0.91	0.91	0.80

Supplementary Figure 3: Cell viability assessment in benign CCD-33CO cells after the combined application of brusatol and irinotecan. (A) Combination with 300 nM brusatol caused a significant decrease in the IC50 value of irinotecan (extra sum-of-squares *F* test). **(B)** Calculated combination indexes for treatment with irinotecan and brusatol confirm additive effects, in contrast to the high degree of synergy shown when this drug combination was used in the CRC cell lines (see Figure 4 in main manuscript for comparison). All graphs display mean data with error bars representing standard deviation. IR = irinotecan.

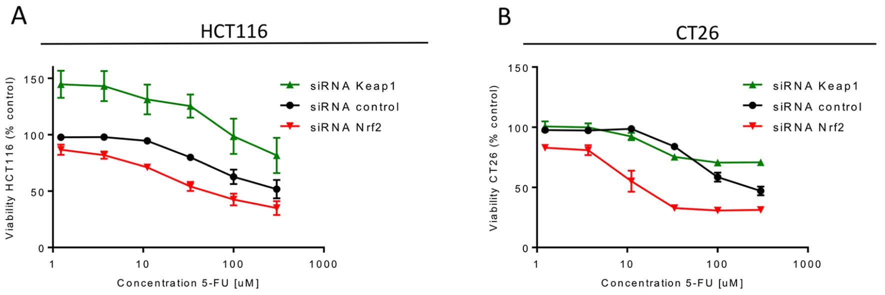

Drug combination	IC50 (uM)	95% confidence interval (uM)	Significance (F test)
5-FU + siRNA Keap1	NC	Not converged	NA
5-FU siRNA control	278.6	204.8 to 379.2	NA
5-FU + siRNA Nrf2	62.6	49.3 to 79.5	***

Drug combination	IC50 (uM)	95% confidence interval (uM)	Significance (F test)
5-FU + siRNA Keap1	NC	Not converged	NA
5-FU + siRNA control	214.3	171.5 to 267.7	NA
5-FU + siRNA Nrf2	21.9	14.5 to 33.3	****

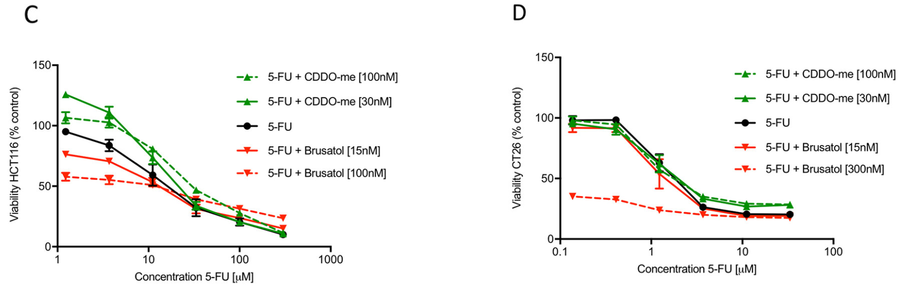

Drug combination	IC50 (uM)	95% confidence interval (uM)	Significance (F test)
5-FU + CDDO-me [100nM]	37.3	30.6 to 45.4	****
5-FU + CDDO-me [30nM]	25.1	17.1 to 36.7	*
5-FU alone	17.9	15.7 to 20.4	NA
5-FU + brusatol [15nM]	12.3	10.4 to 14.7	**
5-FU + brusatol [100nM]	6.7	5.0 to 9.0	****

Drug combination	IC50 (uM)	95% confidence interval (uM)	Significance (F test)
5-FU + CDDO-me [100nM]	3.0	2.0 to 4.5	NS
5-FU + CDDO-me [30nM]	2.8	2.0 to 4.1	NS
5-FU alone	2.2	1.795 to 2.704	NA
5-FU + brusatol [15nM]	1.8	1.3 to 2.4	NS
5-FU + brusatol [300nM]	6.9E-03	2.0E-03 to 3.0E-03	****

E

HCT116 combination indexes scores

		[5-FU]					
[Brusatol]	uM	300.0	100.0	33.0	11.1	3.7	1.23
	0.015	2.58	1.61	0.84	0.91	1.16	1.12
	0.1	4.75	2.73	1.78	1.90	1.82	1.95

F

CT26 combination indexes scores

		[5-FU]					
[Brusatol]	uM	33.0	11.1	3.7	1.23	0.41	0.137
	0.015	2.31	0.83	0.37	0.41	1.10	ND
	0.3	2.35	1.05	0.63	0.45	0.68	0.69

Supplementary Figure 4: Nrf2 inhibition using siRNA significantly increased the cytotoxicity of 5-FU in HCT116 **(A)** and CT26 **(B)** cells as reflected by the decrease IC50 values when compared to treatment with 5-FU alone (extra sum-of-squares *F* test). The cytoprotective effect of overexpression of Nrf2 by KEAP1 inhibition was evident in both cell lines meaning that IC50 could not be calculated due to the blunting of 5FU cytotoxicity. Pharmacological modulation of Nrf2 with brusatol and CDDO-me in combination with 5-FU exhibited the same trends as seen with siRNA transfection in both HCT116 **(C)** and CT26 **(D)** cells. Calculated combination indexes for treatment with 5-FU and brusatol confirm drug synergy in HCT116 **(E)** and CT26 **(F)** cells across some concentrations, but far fewer than seen with irinotecan. Red signifies a higher degree of drug synergy. All graphs display mean data with error bars representing standard deviation. IR = irinotecan.

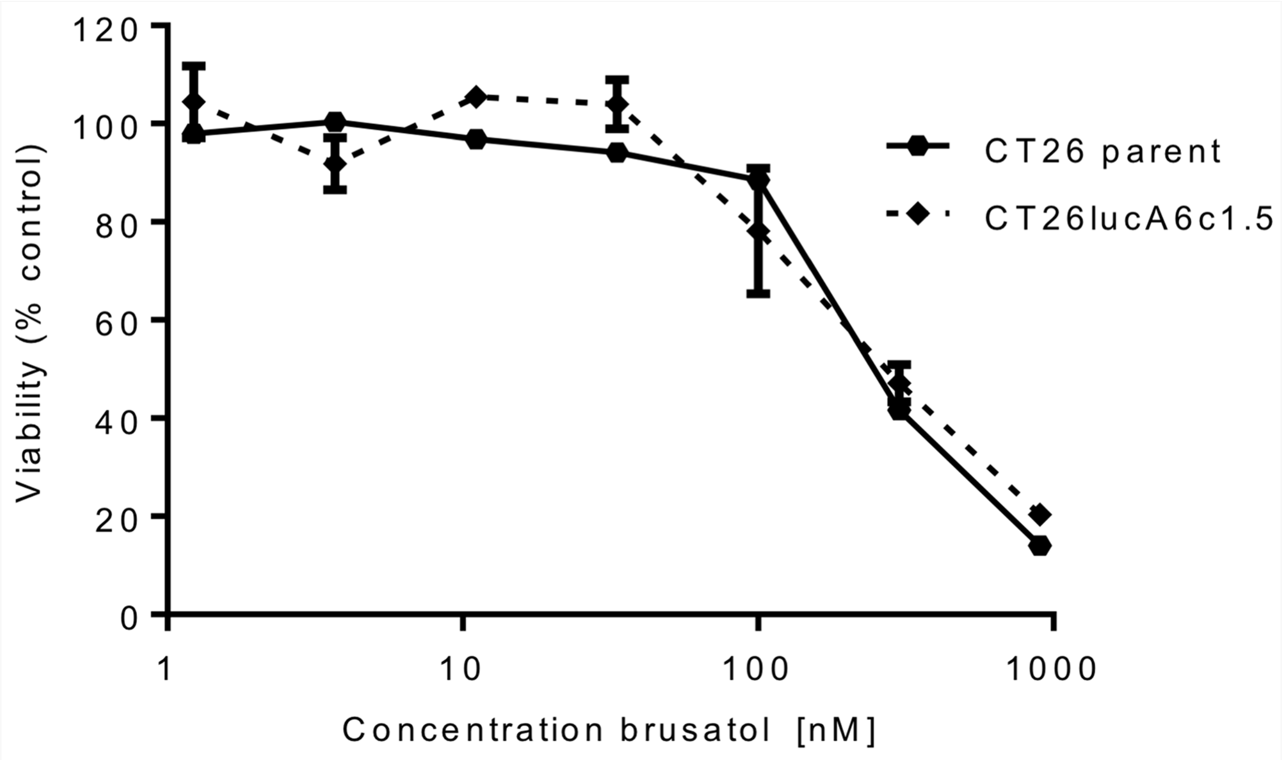

Supplementary Figure 5: Brusatol concentration-response curves demonstrated similar IC50 values in the parent and clonal population (treatment time – 48h; detection by MTS; IC50 same for both data sets=231.8nM (95% CI 200 to 268.6nM, p=0.592 by sum of squares *F*-test).

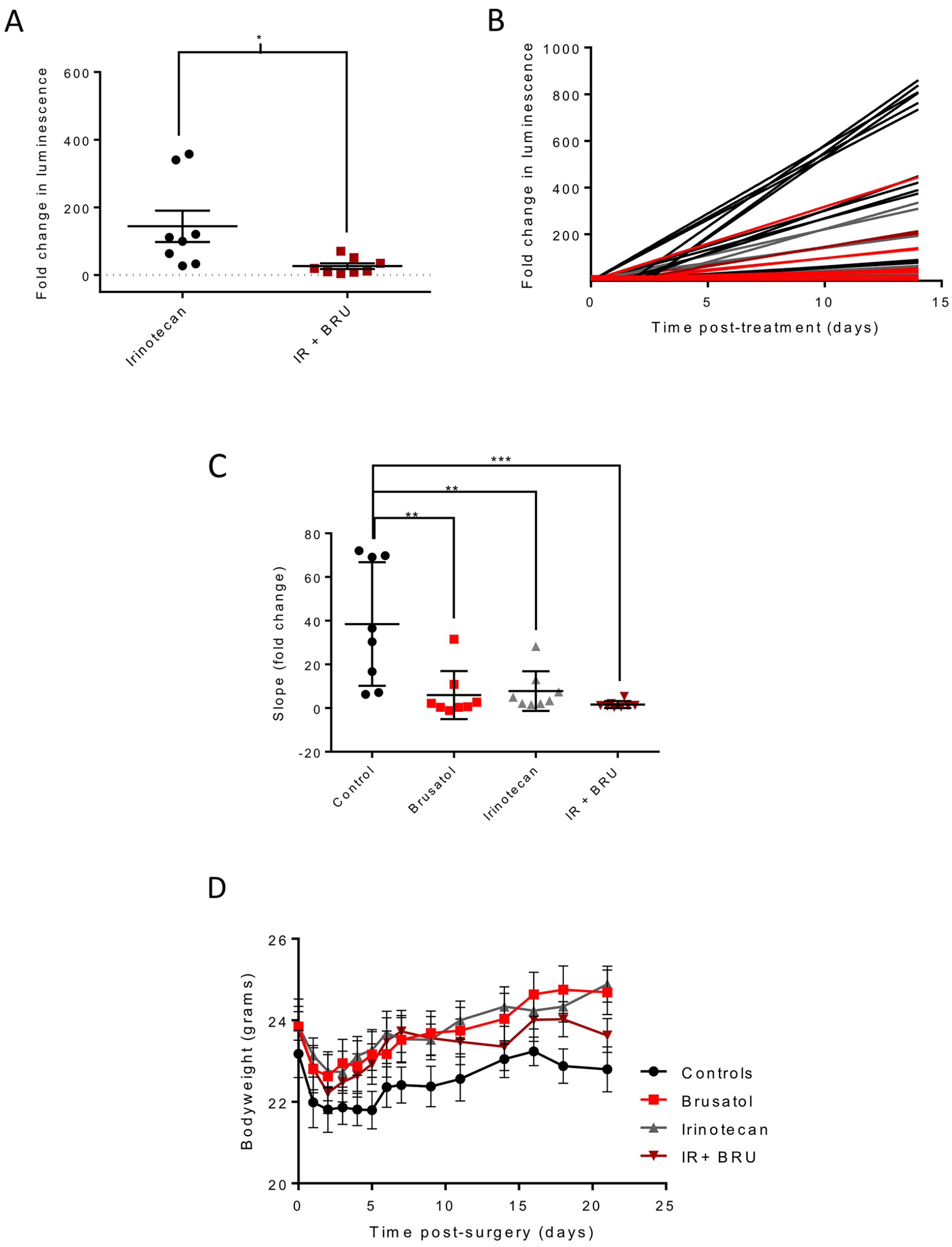

Supplementary Figure 6: **(A)** Fold change in luminescence on the 14th day post-treatment was significantly different in mice on the combination therapy rather than irinotecan alone (unpaired *t*-test with welch correction). **(B)** Graph displays the lines of best fit for fold change in luminescence for each individual mouse, with the slope representing tumour growth rate; colours divide mice by treatment group (black = control, bright red = brusatol, grey = irinotecan and dark red = IR+BRU). **(C)** Scatter plots display the slope values for each individual mouse with the mean +/- SD for each treatment group. All treatments inhibited tumour growth significantly (one-way ANOVA). **(D)** Graph displaying the bodyweight of BALB/c mice orthotopically implanted with the CT26lucA6c cell line over time. Treatment was initiated on day 7 post-surgery. IR = irinotecan, BRU = Brusatol.
